# High-Definition Ultrasound Characterization of Squamous Carcinoma of the Tongue: A Descriptive Observational Study

**DOI:** 10.3390/cancers14030564

**Published:** 2022-01-23

**Authors:** Dario Di Stasio, Marco Montella, Antonio Romano, Giuseppe Colella, Rosario Serpico, Alberta Lucchese

**Affiliations:** 1 Multidisciplinary Department of Medical-Surgical and Dental Specialties, University of Campania “Luigi Vanvitelli”, 80138 Naples, Italy; dario.distasio@unicampania.it (D.D.S.); antonio.romano4@unicampania.it (A.R.); giuseppe.colella@unicampania.it (G.C.); rosario.serpico@unicampania.it (R.S.); 2 Department of Hygiene and Mental Health, University of Campania “Luigi Vanvitelli”, 80138 Naples, Italy; marco.montella@unicampania.it

**Keywords:** OSCC, high-frequency ultrasound, tongue squamous cell carcinoma

## Abstract

**Simple Summary:**

The application of high-frequency ultrasound in the oral cavity for malignant lesions is a growing trend. As with other parts of the body, this method could become routine in the future. With this in mind, this work aimed to characterize squamous tumors of the tongue ultrasonographically and added additional descriptive elements to the current literature.

**Abstract:**

High-definition ultrasonography is a diagnostic tool that uses sound echoes to produce images of tissues and organs. In the head and neck region, ultrasounds have been used to diagnose different types of lesions. The intraoral approach was shown to be a real-time, non-invasive way to characterize oral lesions. The tongue is the most often examined region because of its accessibility. This observational study aimed to describe the qualitative characteristics of tongue squamous cell carcinoma images obtained with high-definition intraoral ultrasound by comparing them with the corresponding histopathological sample. Twenty patients were enrolled in this study. The scans of the lesions were carried out with an 18 MHz linear ultrasound probe following the long axis of the lesion. For each lesion, five frames were selected, on which descriptive analysis was performed. A histological sample was taken and then compared to the ultrasonographic acquisition. The sonographic appearance of the tissue layers has a good correlation between ultrasound and histological morphology, and it was easy to distinguish the tumor from the homogenous composition of the tongue tissues. Furthermore, a correlation between the structure by section and pattern of tumor margin features by ultrasound was obtained. Intraoral ultrasonography appears to be a promising technique in the non-invasive characterization of tongue squamous cell carcinoma. Further studies will be needed to improve the technique in terms of ergonomics and repeatability.

## 1. Introduction

The most common malignant tumor of the oral cavity is squamous cell carcinoma (OSCC), which often arises from precursor lesions [1]. To date, the gold standard for its diagnosis is still a biopsy for histopathological examination; however, novel imaging techniques are under evaluation to shorten and improve the diagnostic pathway [2,3,4,5].

Ultrasonography (US) has been used in various branches of medicine such as gynecology, gastroenterology, cardiology, and angiology. In the head and neck region, it has been used to detect salivary glands abnormalities and malignancy [6,7]. This technique has been shown to be useful for several purposes: from studying periodontal tissues to the clinical and presurgical characterization of benign and malignant lesions of the oral mucosa [8,9,10].

Shintani et al. were the first to decipher the sonographic characteristics of the healthy oral mucosa. They identified a homogeneous ultrasonographic pattern of the healthy tongue; the normal buccal mucosa presented a homogeneous pattern, too, with a hyperechoic aspect due to the thick cortical bone of the mandible, while some acoustical shadowing characterized a regular pattern of the mouth floor due to the sublingual glands [11]. These authors used technologically very distant equipment from those used in the high-frequency US, with emission of only 7.5 MHZ. With the progress of technology, some recent ex vivo and in vivo studies suggest that the high-frequency US acquired the capacity to discern tissue layers on the basis of image contrast. This contrast results from acoustic backscatter, directly proportional to the change in density found in the boundaries between tissue layers. As a result, less dense fat tissue layers appear hypoechoic, whereas denser tissue layers such as muscle and connective tissue appear hyperechoic on images [12]. The tongue is the most often examined region because it is easily accessible compared to other oral cavity sites. Furthermore, it is easy to distinguish the tumor from the homogenous muscular composition of the tongue while other disturbing anatomical structures are absent [13,14].

On the basis of the data in the literature, this descriptive qualitative study aims to verify if the images obtained with the US in real time could overlap the histological images and if it is possible to obtain, from this comparison, helpful information to better define the structures observed in high-definition US.

## 2. Materials and Methods

Patients were recruited at the Oral Pathology Unit at the University of Campania L. Vanvitelli, Naples, Italy, from December 2018 to March 2020 after giving written informed consent.

Twenty consecutive patients reporting oral lesions of the mobile tongue clinically suspected of malignancies were considered before undergoing conventional diagnostic protocols (biopsy and histopathological assessment). Patients’ clinical data are reported in Table 1. For comparison purposes, scans of the contralateral healthy mucosa were performed. All lesions and sites were at first analyzed with US imaging, and then they were biopsied. The US images’ real-time acquisition and interpretation were performed at the end of clinical examination. Tongue lesions were classified on the basis of the clinical presentation in ulcerated and nodular.

**Figure 1:**
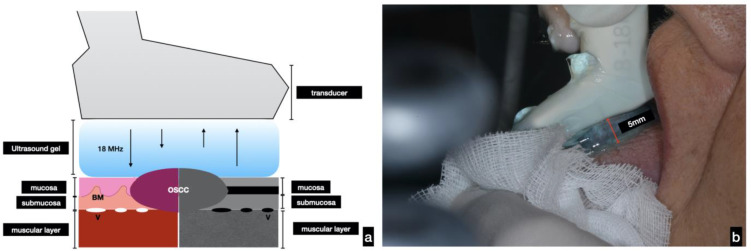
(**a**) Schematic representation of intraoral US examination and imaging acquisition. In the lower part of the figure, a comparison of the ultrasound imaging (right) with pathological features (left) can be observed; (**b**) during the US evaluation, the probe was placed, following a non-contact technique, at a 5 mm distance to the tissues examined.

The images were acquired with the GE Logiq-e R7 device with a 15 LCD monitor, with a resolution of 1024 × 768. The transducer used was an L8-18i-D “hockey” probe, with a frequency range of 5–18 MHz and an axial field of view from 0.4 mm to 2 cm and the side from 0.7 mm to 2 cm. The examination was performed after applying ultrasound gel on the area to be scanned. A fixed distance of about 5 mm between the lesion and the probe was set (Figure 1). The focus was standardized. For each lesion, five frames were selected, on which measurements and descriptive analysis were performed. For comparison purposes, scans of the contralateral healthy mucosa were performed. All images were cataloged and analyzed using Horos imaging software (ver. 3.2.1) [15].

After excision, all samples were fixed in 10% neutral buffered formalin and embedded in paraffin. Macroscopically, all the lesions were sliced along to the major axis of the lesion to be comparable with the ultrasonographic acquisition. From the inclusions thus obtained, 5 µm sections were cut and stained in hematoxylin and eosin for morphological evaluation and diagnosis. All the OSCCs were defined histologically according to WHO Classification of Tumours, 8th Edition (2017) [16]. Grading was assessed using the conventional 3-tier system: well-differentiated (G1), moderately differentiated (G2), and poorly differentiated (G3).

## 3. Results

A total of 20 patients (6 women and 14 men; mean age 65.3 ± 13.22 y.o; age range 36–82 years) with a single OSCC lesion of the tongue were considered. A total of 10 ulcerated and 10 nodular OSCC were collected and analyzed. Histological features of all lesions are summarized in Table 1.

### 3.1. Healthy Tongue Features

**Figure 2:**
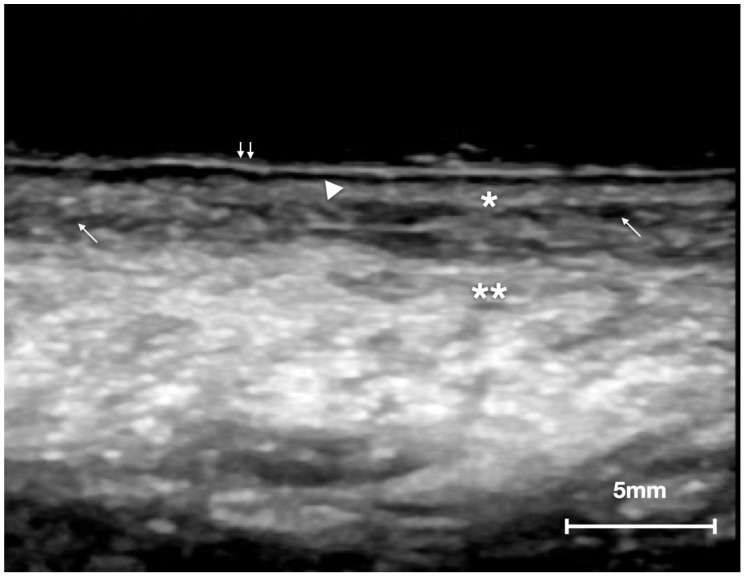
US image of normal tongue: (double arrow) epithelium; (arrow head) epithelial–connective junction—rete ridges; (*) submucosa; (**) muscular layer; (arrow) vessels.

According to the patients analyzed, the ultrasound structure of the healthy contralateral side of the tongue appeared as follows from the surface to the deeper layers (Figure 2):

(a) A well-defined continuous hyperechoic band that corresponded to the epithelial layer.
(b) An anechoic line with a thickness similar to the epithelium above, representing the short, blunt rete ridges. This layer consisted of the oral mucosa limit since it is impossible to identify the basement membrane for resolution limits of all ultrasound equipment (even at higher frequencies).
(c) A homogeneous, relatively hypoechoic band of connective tissue, where submucosa merged and intersected with the ventral muscle bundles of the tongue.
(d) An alternation of hypoechogenic, hyperechoic, and anechoic areas configuring striated muscle bundles that recall the histological fascicular features. A very slight acoustic enhancement was present. This layer represented the thickest portion of the ultrasound image, and within this area, it was also possible to observe the vessels of medium and large caliber.

### 3.2. Ultrasonographic Appearance of Tongue OSCC

Oral cancer of the tongue appeared on the US imaging as a well-defined, non-homogeneous hypoechoic area compared to surrounding tissues. What seems clear is the loss of the US features of normal oral mucosa described above in the areas where the carcinoma infiltrated the tongue. The ultrasound image seemed to replicate the exact shape of the macroscopic section. Furthermore, the lesions we analyzed did not show ultrasound artifacts such as acoustic shadowing. In seven lesions, however, a moderately hypoechoic area underneath the lesion was present. Those areas histologically corresponded to the different densities of the muscular layer. This phenomenon could have been due to compressive/infiltrative features of some neoplasm pattern of infiltration with “pushing border” instead of “infiltrative or single cells” border.

**Figure 3:**
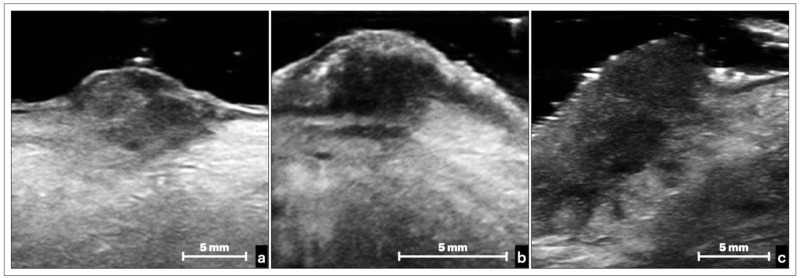
Ultrasound images of three tongue OSCCs, histologically diagnosed as well-differentiated—G1 (**a**), moderately differentiated—G2 (**b**), and poorly differentiated—G3 (**c**). On a careful examination, it is possible to appreciate an increasing hypoechogenic gradient from the G1 to G3 lesion (**a**,**c**), a more significant irregularity of the more “expansive” infiltration borders in the more differentiated lesions (G1–G2), and more “infiltrative” borders in the poorly differentiated lesions (G3).

Moreover, from the ultrasound analysis of the lesions, differences can be noted on the basis of the histopathological grading: in the lower-grade lesions (G1 and G2), the margins appeared more defined and expansive; in the high-grade lesions (G3), the deep edges of the lesion appeared less definable (Figure 3). Furthermore, the high-grade lesions appeared more markedly hypoechoic (Figure 3c), while in grade G1 and G2 lesions, there was an inhomogeneous picture with mixed iso-hyperechoic areas in the context of the lesion (Figure 3a,b). These characteristics did not show a clear difference between low- and medium-grade lesions, while the distinction with high-grade lesions appeared clearer.

#### 3.2.1. Ulcerated OSCC

**Figure 4:**
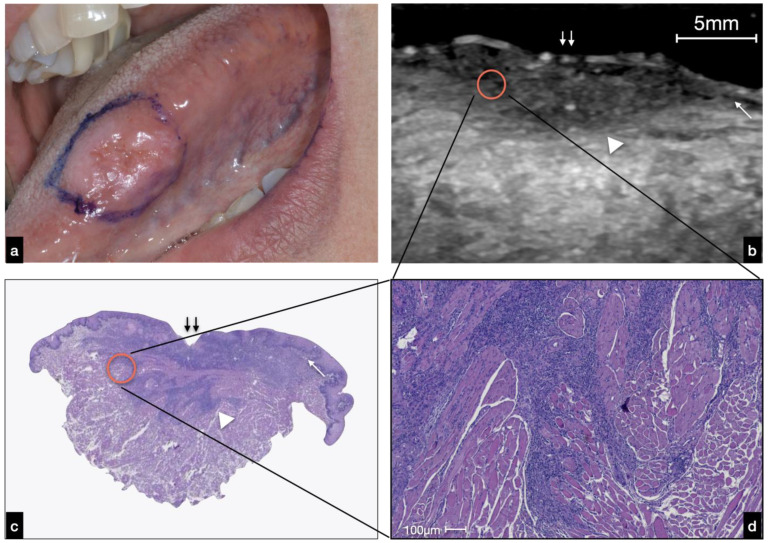
(**a**) Clinical presentation of an ulcerated OSCC of the left tongue margin. (**b**) US evaluation and histological analysis showing an interruption of the covering mucosa (double arrow) at the area of ulceration; at the peripheral region, the regular mucosa interface is still visible (white arrow). The limit between normal tissue and neoplasm is not well defined in this case (white arrowhead) due to the infiltrative pattern of this OSCC as enlightened in the red circle (**c**). (**d**) Strands of neoplastic cells intermingled with muscle bundle are visible at higher magnification.

In lesions ulcerated by OSCC, we noted a break in the epithelium. This fragmentation increased, getting closer to the center of the lesion. The lesion developed in-depth, and it was possible to distinguish it (in terms of echogenicity) by the surrounding tissues. The relationships in the core of the lesion between the ultrasound recognizable structures were altered and disorganized (Figure 4).

#### 3.2.2. Nodular OSCC

**Figure 5:**
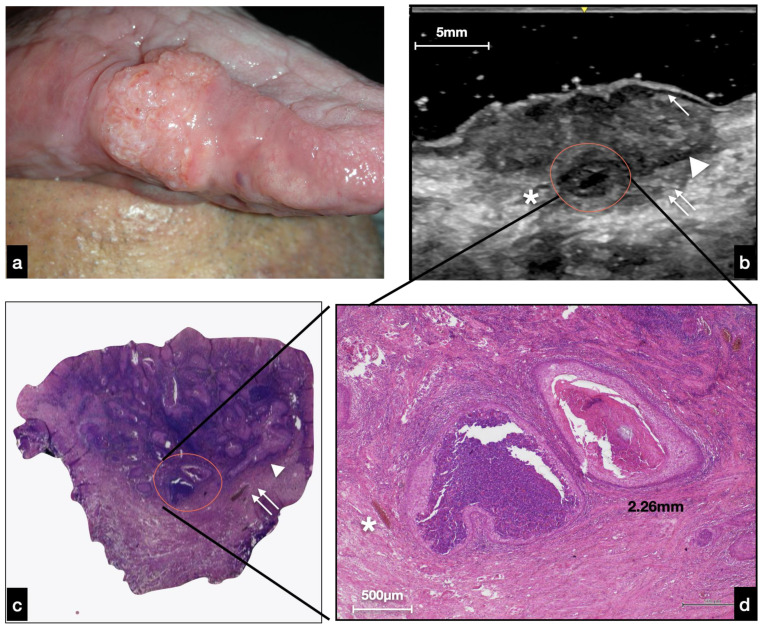
(**a**) Clinical presentation of a nodular OSCC of the right tongue margin. (**b**) US evaluation: at the lateral edges of the lesion, the hypoechoic limit of the normal epithelium is still visible (white arrow); the neoplasm is well delimited by a homogeneous hypoechoic line (white arrowhead). At the bottom of the lesion, there is a non-homogeneous hypoechoic area (double white arrow) histologically corresponding to the “peripherally pushed” muscle; a roundish, well delimited anechoic area (red circle) was found, histologically corresponding to a neoplastic abscess. A small vessel nearby the abscess was identified (white asterisk) (**b**–**d**).

The lesions analyzed in this study appeared as heterogeneously hypoechoic and well-defined areas. As shown in Figure 5, the lesion’s periphery at the superficial level can preserve the epithelial–connective interface until it is completely lost as it gets closer to the lesion (Figure 5b). However, below them, there is a well-defined hypoechoic tissue that corresponded to tumor tissue in comparison with the histology. This deep compound of the neoplasm determined the exophytic clinical feature of the mass (Figure 5a). The hypoechoic mass developed deeply and was distinguishable from the surrounding relatively hyperechoic muscle tissue with an invasive basal border.

Given that the dimensional accuracy of the ultrasound examination was exceptional, this technique could also allow for the recognition of structures visible only at the histological level (microscopic section). For example, a well-defined anechoic area of about 2.3 mm in diameter, surrounded by a border of relatively hypoechoic tumor tissue, was found (Figure 5b). This morphological detail observed at ultrasound examination histologically corresponded to deep-located neoplastic nests, composed peripherally of atypical squamous cells and centrally by granulocytes (abscess) and keratinocytes sheets, measuring about 2.2 mm in diameter (Figure 5c,d).

## 4. Discussion

This ultrasound analysis is still in the definition phase, and therefore it seems mandatory to conduct a comparative analysis with the ultrasound study of other districts and other neoplasia.

The ultrasound study of neoplasms is used for many organs and tissues, from skin [17], tyroid [18], and salivary glands [19], to breasts [20], lungs [21], and the pancreas [22]. It is obvious how the differences in the individual anatomical areas translate into differences in the ultrasound appearance, such that it is necessary to use different evaluation parameters compared to those used for routine.

In this sense, some biases that limit the comparison must be identified: (a) the qualitative differences between our probe and those commonly used (less performing, with different Hz), (b) intrinsic differences in the anatomical area under study, and (c) intrinsic differences in the histology of the tumor under study.

Considering these limitations, moving in search of univocal evaluation parameters, the most natural comparison appears with the skin district, compared to all the other parenchyma studied, since the skin shares a similar coating epithelium with the oral cavity, an equal subdivision into “layers”. The most consistent data in the literature concern melanoma, in search of an ultrasound Breslow thickness rather than in the characterization of squamous lesions [23], used in pre-operative assessment and post-operative follow-up, to evaluate the three-dimensional size of a tumor relationship to surrounding structures [24]. In any case, due to its histological features, OSCC could be compared with squamous cell carcinoma (SCC) and squamous cell basalioma (BCC) of the skin. According to the data reported in the literature, at US imaging, BCC appeared as well-defined, oval, or slightly irregular, hypoechoic lesions that usually present hyperechoic spots (representing the neoplasm nests).

In contrast, SCC presents as a heterogeneously hypoechoic lesion with irregular borders, lack of hyperechoic spots, and involves the deeper layers [25,26]. From the data reported in the present study, the OSCC has specific characteristics concerning the grading and invasiveness detected by the pathological analysis in the US. The lesions appeared to be well defined and easily recognizable compared to the surrounding tissues, but the deep layers may be well delimited in the lesions with little invasiveness or more nuanced in the lesions with more significant characteristics of aggressiveness. The intraoral US has been seen to be particularly useful in determining the interface between tumors and the surrounding myo-architecture at the deep margin [27]. The ultrasonographic pattern of most of the invasive carcinomas was a relatively well-defined hypoechoic lesion, while the pattern of the surrounding tissues was homogeneous echogenic [11,28]. However, some authors reported that a hyperechoic image was visualized with posterior sonography attenuation when an ulcerated area was present due to air interpositioning [28]. These features were not found in the images obtained and analyzed in the present study.

Concerning the possibility to distinguish the different tumor grades by the US examination, the comparison with the confirmed histological picture could facilitate the interpretation of the ultrasound images. There are no data in the literature regarding the hypo-echogenicity observed in high-grade lesions, and the series currently under examination is too small for statistically significant representativeness. However, it would be possible to hypothesize that the keratinization, observed at the US as “hyperechoic” more present in low-grade lesions, moisturizes an overall inhomogeneous ultrasound image. On the other hand, high-grade lesions, which tend to have little or no keratinizing effects, show a more hypoechoic image, perhaps by greater cellularity. However, these speculations require further study and with a larger patient cohort.

## 5. Conclusions

The data presented in this study, although preliminary, are promising and reinforce the data already present in the literature on the use of US in the oral cavity [9,29]. However, it is necessary to highlight the limits of the technique represented by the interpretation of the images themselves and by the ability of the operator. For example, the probe must be held gently but with a firm hand. The contact of the transducer with the skin should be as smooth as possible to avoid compression of the anatomical structures below and incorrect measurements. Excessive pressure of the transducers may modify the image, particularly in the uppermost portion of the FOV, flattening the abnormalities and causing an under-estimation of the OSCC thickness [23]. In this context, the importance of new studies aimed at determining main tumor characteristics (tumor thickness, depth of invasion, and staging) that can guide the clinician in vivo and real-time diagnosis and the management of the cancer patient.

## Figures and Tables

**Table 1:** Demographic, clinical, and histopathological data.

Patient	Age	Gender	Clinical Features	Tongue Subsite	Histopathology
1	73	F	ulcerated	left margin	OSCC—G2
2	69	M	nodular	right margin	OSCC—G2
3	36	M	ulcerated	left margin	OSCC—G2
4	79	M	ulcerated	dorsum/right margin	OSCC—G2
5	50	M	ulcerated	right margin	OSCC—G1
6	82	F	ulcerated	right margin	OSCC—G2
7	79	M	nodular	ventral/left margin	OSCC—G1
8	79	F	nodular	left margin	OSCC—G1
9	76	M	nodular	dorsum/left margin	OSCC—G2
10	47	M	ulcerated	ventral/right margin	OSCC—G1
11	73	M	nodular	right margin	OSCC—G1
12	73	F	nodular	dorsum/right margin	OSCC—G1
13	57	M	nodular	right margin	OSCC—G1
14	78	M	ulcerated	left margin	OSCC—G1
15	60	M	ulcerated	left margin	OSCC—G1
16	68	M	ulcerated	left margin	OSCC—G1
17	45	M	nodular	right margin	OSCC—G1
18	61	F	ulcerated	left margin	OSCC—G2
19	70	M	nodular	left margin	OSCC—G3
20	51	F	nodular	right margin	OSCC—G1

## Data Availability

Data sharing not applicable.

## Abbreviations

US	ultrasound
OSCC	oral squamous cell carcinoma
SCC	squamous cell carcinoma
BCC	squamous cell basalioma
BM	basal membrane
V	vessel
